# Exploring the effect of primary tumor sidedness on therapeutic efficacy across treatment lines in patients with metastatic colorectal cancer: analysis of FIRE-3 (AIOKRK0306)

**DOI:** 10.18632/oncotarget.22396

**Published:** 2017-11-11

**Authors:** Dominik Paul Modest, Sebastian Stintzing, Ludwig Fischer von Weikersthal, Thomas Decker, Alexander Kiani, Ursula Vehling-Kaiser, Salah-Eddin Al-Batran, Tobias Heintges, Christoph Kahl, Gernot Seipelt, Frank Kullmann, Werner Scheithauer, Markus Moehler, Julian Walter Holch, Jobst Christian von Einem, Swantje Held, Volker Heinemann

**Affiliations:** ^1^ Department of Medicine III, University Hospital, LMU Munich, Munich, Germany; ^2^ German Cancer Consortium (DKTK), German Cancer Research Centre (DKFZ), Heidelberg, Germany; ^3^ Gesundheitszentrum St. Marien, Amberg, Germany; ^4^ Oncological Practice, Ravensburg, Germany; ^5^ Medizinische Klinik IV, Klinikum Bayreuth, Bayreuth, Germany; ^6^ Oncological Practice, Landshut, Germany; ^7^ Department of Hematology and Oncology, Krankenhaus Nordwest Frankfurt/Main, Frankfurt, Germany; ^8^ Department of Medicine II, Städtisches Klinikum Neuss, Neuss, Germany; ^9^ Haematology and Oncology, Staedtisches Klinikum Magdeburg, Magdeburg, Germany; ^10^ Oncological Practice, Bad Soden, Germany; ^11^ Department of Medicine I, Klinikum Weiden, Weiden in der Oberpfalz, Germany; ^12^ Department of Internal Medicine I & Comprehensive Cancer Center, Medical University Vienna, Vienna, Austria; ^13^ Medical Department 1, Johannes-Gutenberg Universität Mainz, Mainz, Germany; ^14^ University Cancer Center Frankfurt/Mainz and German Cancer Consortium (DKTK), Heidelberg, Germany; ^15^ ClinAssess GmbH, Leverkusen, Germany

**Keywords:** colorectal cancer, tumor sidedness, EGFR antibody, bevacizumab, sequential therapy

## Abstract

**Purpose:**

To assess the impact of primary tumor sidedness on outcome of patients with metastatic colorectal cancer (mCRC) across treatment lines.

**Patients and Methods:**

Patients of the FIRE-3 trial (initial FOLFIRI plus either cetuximab or bevacizumab) were separately evaluated according to primary tumor site differentiating left-sided (LPT) from right-sided primary tumors (RPT). Efficacy (i.e. progression-free survival (PFS2nd) and overall survival (OS2nd) of second-line therapy) was evaluated by Kaplan-Meier method and compared by log rank test as well as Cox regression analyses. All analyses were also reported according to drug sequences.

**Results:**

411 of 592 patients (69%) with KRAS exon 2 wild-type tumors received 2nd-line therapy has and had available information on primary tumor location, of those 309 patients (75%) presented with LPT. In patients with LPT, PFS2nd was markedly longer than in patients with RPT (6.0 months [95% CI 5.5-6.5] versus 3.8 months [95% CI 2.5-5.2], hazard ratio: 0.61 [95% CI 0.47-0.78], P<0.001). Differences in PFS2nd between study-arms were evident in patients with LPT, but not in patients with RPT (Cox model interaction test, P=0.12). Consistent observations were also made for OS2nd.

**Conclusion:**

This retrospective analysis of FIRE-3 indicates that efficacy of second-line therapy was significantly greater in patients with left-sided tumors as compared to right-sided tumors. This difference was driven by superior activity of second-line regimens of the initial cetuximab-arm as compared to the initial bevacizumab-arm in left-sided tumors. Our observations confirm the strong prognostic value of primary tumor location in second-line therapy of mCRC.

## INTRODUCTION

Primary tumor sidedness has been identified as prognostic and predictive information in patients with metastatic colorectal cancer (mCRC) [1–4]. The predictive relevance of primary tumor sidedness has been specifically demonstrated with regard to monoclonal antibodies targeting the epidermal growth factor receptor (EGFR-mAb) [1, 5]. Although a definite biologic explanation is not yet available, primary tumor sidedness can be used for decision making in mCRC. As currently available data are primarily derived from studies investigating first-line treatment of mCRC, they can also be applied in the context of FIRE-3.

Results from several recently published studies suggest that survival times >30 months can be expected in patients with KRAS/RAS wild-type mCRC [6–8] fit for intensive therapy. Most of these patients are likely to be treated in various treatment lines (and modalities) beyond inital therapy. Unfortunately, frequency and efficacy of therapies beyond 1^st^ line, although impacting on outcome, are rarely documented in most clinical studies. This limitation is specifically important to patients with left-sided mCRC that may live years beyond the first treatment [1, 5]. Consecutively, it remains unclear to which extent primary tumor sidedness plays a role with regard to efficacy of first-line versus later-line treatment. Moreover, only very limited and potentially conflicting data are available from studies investigating second-line treatment of mCRC involving EGFR-targeted therapy [9, 10].

FIRE-3 (AIOKRK0306) randomized patients with KRAS exon 2 wild-type mCRC into either FOLFIRI plus cetuximab (arm A) or FOLFIRI plus bevacizumab (arm B) as first-line therapy. The differences in overall survival were not associated with similar results in classical early endpoints like response rate (primary endpoint) or progression-free survival [11]. Nevertheless, differences in depth of response (DpR) and efficacy of second-line regimens – both aspects favoured the cetuximab-arm - provide possible explanations for the observed benefit in overall survival [7, 12]. In FIRE-3, the documentation of treatment and efficacy across several lines allows for analyses of the impact of primary tumor location on efficacy of first and second-line therapy. The present analysis now focuses on primary tumor sidedness in relation to frequency of subsequent agents, death-rates according to treatment line, and treatment efficacy in second-line therapy. Specifically, study data may help to generate hypotheses on the role of second-line therapy for the observed effects of primary-tumor sidedness on outcome. In addition, the effects of distinct sequences of targeted agents in LPT versus RPT mCRC is explored in this manuscript.

To the best of our knowledge, this is the first investigation evaluating the effect of tumor sidedness on treatment outcome with regard to different targeted agents and across treatment lines. This manuscript represents a post-hoc analysis and should be interpreted as such.

## RESULTS

Of 592 patients with *KRAS* exon 2 wild-type tumors in the ITT-population, 414 (70%) patients received second-line therapy. In three of these 414 patients, the exact information on localisation of the primary tumor is unknown. Therefore, this manuscript focuses on 411 patients (309 LPT, 102 RPT) with KRAS exon 2 wild-type mCRC, documented second-line therapy and available information on primary tumor location. Of those 411 patients, 238 patients had centrally tested RAS/BRAF wild-type tumors (187 patients with LPT and 51 patients with RPT). Thirty-four patients with BRAF mutant tumors were also part of the analysis set (18 patients with LPT and 16 patients with RPT). The clinical characteristics of the 411 patients with second-line therapy and defined tumor location are summarized in Table 1.

**Table 1:** Baseline characteristics of the second-line population

Characteristics	Left-sided primary (N=309)				Right-sided primary (N=102)			
	FOLFIRI + Cetuximab		FOLFIRI + Bevacizumab		FOLFIRI + Cetuximab		FOLFIRI + Bevacizumab	
	N=170	100%	N=139	100%	N=40	100%	N=62	100%
**Age, median**	63	-	64	-	67	-	65	-
**Age ≤ 65**	101	59.4	75	54.0	18	45.0	32	51.6
**Age ≤ 70**	134	78.8	111	79.9	30	75.0	46	74.2
**male**	123	72.4	88	63.3	22	55.0	33	53.2
**female**	47	27.6	51	36.7	18	45.0	29	46.8
**ECOG 0**	100	58.8	72	51.8	17	42.5	31	50.0
**ECOG 1**	67	39.4	66	47.5	22	55.0	30	48.4
**ECOG 2**	3	1.8	1	0.7	1	2.5	1	1.6
**Leucoc≥ 8/nl**	72	42.4	58	41.7	16	40.0	23	37.1
**AP ≥ 300 U/L**	23	13.5	19	13.7	3	7.5	10	16.1
**Colon primary**	82	48.2	69	49.6	40	100.0	62	100.0
**Rectum primary**	82	48.2	62	44.6	-	-	-	-
**(Colon + Rectum)**	6	3.5	8	5.8	-	-	-	-
**Liver metastases**	143	84.1	118	84.9	33	82.5	49	79.0
**Lung metastases**	75	44.1	58	41.7	12	30.0	20	32.3
**Lymph node mets.**	61	35.9	37	26.6	11	27.5	29	46.8
**Peritoneal mets.**	9	5.3	8	5.8	6	15.0	7	11.3
**Other mets.**	37	21.8	32	23.0	6	15.0	15	24.2
**Liver-limited mets.**	55	32.4	46	33.1	16	40.0	19	30.6
**Lung-limited mets**	6	3.5	8	5.8	-	-	2	3.2
**1-organ disease**	66	38.8	57	41.0	21	52.5	25	40.3
**2-organ disease**	63	37.1	55	39.6	12	30.0	23	37.1
**3-organ disease**	29	17.1	22	15.8	5	12.5	5	8.1
**4-organ disease**	9	5.3	5	3.6	2	5.0	6	9.7
**5-organ disease**	2	1.2	-	-	-	-	2	3.2
**adjuvant chemo**	40	23.5	30	21.6	8	20.0	9	14.5
**Primary resected**	135	79.4	117	84.2	38	95.0	56	90.3
**Prior radiation therapy**	28	16.5	23	16.5	1	2.5	-	-

Characteristics were recorded as baseline-assessment of FIRE-3 before start of first-line treatment; ^*^ECOG=Eastern Cooperative Oncology Group

### Patients and treatment lines

**Figure 1:**
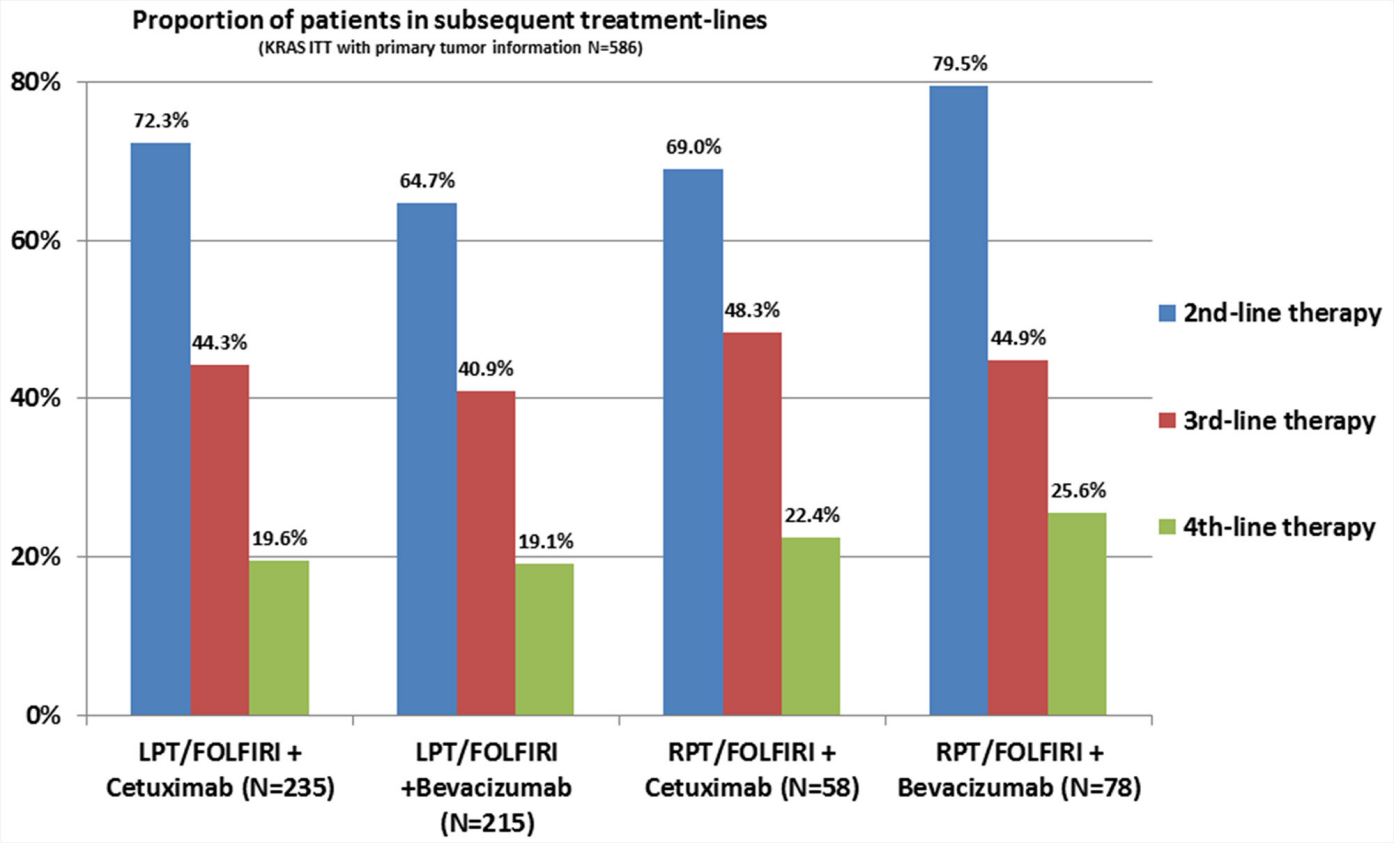
Proportion of patients in subsequent treatment line LPT= left-sided primary tumor; RPT=right-sided primary tumor.

**Figure 2:**
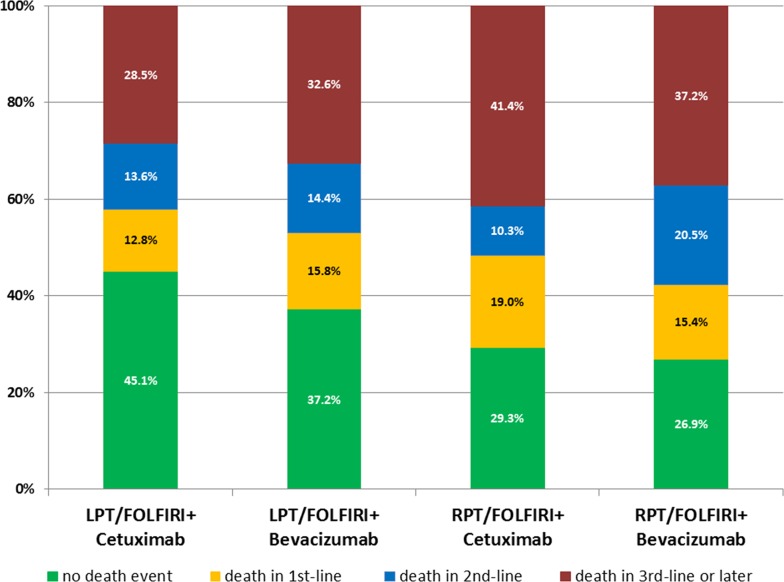
Deaths according to treatment lines in FIRE-3 LPT= left-sided primary tumor; RPT=right-sided primary tumor.

The frequency of second-line therapy according to study arms and primary tumor location ranged from 64.7% (LPT, am B) to 79.5% (RPT, arm B) as shown in Figure 1. The analysis of deaths according to different treatment lines revealed the highest 1^st^-line death rate in patients with RPT receiving cetuximab (19%) and the lowest rate in patients with LPT also receiving cetuximab (12.8%). The first-line death rate in bevacizumab-treated patients was comparable between left- and right-sided mCRC. Details concerning also 2^nd^-line and further-line rates are summarized in Figure 2.

### Second-line use of agents according to primary tumor sidedness and study arm

As in previous reports [12], comparable frequencies of oxaliplatin, fluoropyrimidines and other respective other antibody (−class) were used in both arms of FIRE-3 if primary tumor sidedness was taken into account. Details are summarized in Table 2. In second-line therapy, the following antibody-sequences were observed: in patients with initial cetuximab-based therapy (arm A), 100 patients (33.7% of the initial 297 patients in the intent-to-treat population) received VEGF-targeted second-line regimens. Of those, 84% were attibuted to LPT and 16% to RPT. Among patients with initial bevacizumab-therapy (arm B), 89 (30.2% of the initial 295 patients in intent-to-treat population) patients received second-line regimens containing EGFR-targeted antibodies. Of those, 67.4% were attributed to LPT and 32.6% to RPT.

**Table 2:** Post-study treatment based on the second-line population

Substances	Left-sided primary tumors				Right-sided primary tumors			
	FOLFIRI + Cetuximab		FOLFIRI + Bevacizumab		FOLFIRI + Cetuximab		FOLFIRI + Bevacizumab	
	N=170	100%	N=139	100%	N=40	100%	N=62	100%
**Fluoropyrimidine**	165	97.1	128	92.1	39	97.5	57	91.9
**Irinotecan**	71	41.8	68	48.9	10	25.0	24	38.7
**Oxaliplatin**	131	77.1	112	80.6	33	82.5	45	72.6
**Anti-EGFR antibody**	70	41.2	105	75.5	20	50.0	49	79.0
**Anti-VEGF antibody**	113	66.5	38	27.3	25	62.5	15	24.2

Table lists all recorded subsequent substances in patients that received at least second-line therapy, independent from the treatment-line in which it was used. Only the most frequent subsequent substances are listed.

### Prognostic role of primary tumor sidedness for second-line therapy

**Figure 3:**
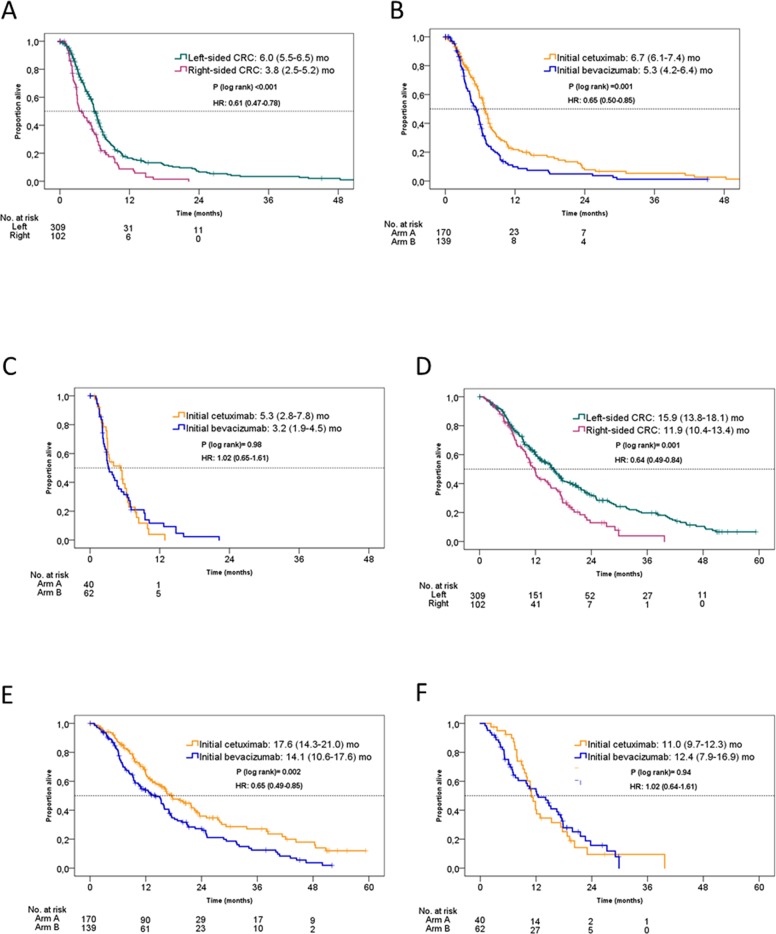
Kaplan-Meier estimates of PFS2nd and OS2nd in the FIRE-3 KRAS exon 2 wild-type population according to initial study arm and primary tumor location **(A)** PFS2nd according to tumor location; **(B)** PFS2nd in patients with left-sided primary tumor according to initial study arm; **(C)** PFS2nd in patients with right-sided primary tumor according to initial study arm; **(D)** OS2nd according to tumor location; **(E)** OS2nd in patients with left-sided primary tumor according to initial study arm; **(F)** PFS2nd in patients with right-sided primary tumor according to initial study arm. arm A= initial FOLFIRI plus cetuximab. arm B= initial FOLFIRI plus bevacizumab.

Objective response to second-line therapy was observed in 68 of 309 patients (22.0%) with LPT (21.2% in the prior cetuximab arm, 23.0% in the prior bevacizumab arm. In patients with RPT, 14 of 102 patients (13.7%) achieved objective response in second-line treatment (10.0% in the prior cetuximab arm, 16.1% in the prior bevacizumab arm). In patients with LPT, PFS2nd was markedly longer than in patients with RPT (6.0 months [95% CI 5.5-6.5] versus 3.8 months [95% CI 2.5-5.2], hazard ratio: 0.61 [95% CI 0.47-0.78], P<0.001). However, the choice of targeted therapy in 1^st^-line treatment (cetuximab vs. bevacizumab) influenced duration of PFS 2^nd^ only in patients with LPT, favoring the initial-cetuximab arm. No difference in outcome between the initial study-groups was observed in patients with RPT and second-line therapy. A Cox model interaction test of primary tumor sidedness and prior treatment arm for PFS 2^nd^ showed a strong trend for an interaction (P=0.12). Consistent observations were made for OS2nd (Figure 3). Tables 3 and 4 contain univariate as well as multivariate analyses of PFS2nd and OS2nd for various prognostic factors. In both, univariate analysis and multivariate analyses, primary tumor location and study arm influenced outcome significantly.

**Table 3:** Univariate analysis of PFS2nd and OS2nd

Parameter	Factor	Hazard ratio	95% CI	P-value
PFS2nd	FIRE-3 treatment arm (bevacizumab vs. cetuximab)	0.68	0.55-0.85	0.0008
	ECOG (0 vs. 1 vs. 2)	0.89	0.72-1.10	0.2721
	Age (years)	1.00	0.99-1.02	0.6042
	Sex (male vs. female)	0.89	0.71-1.13	0.3334
	Primary tumor location (left vs. right)	1.64	1.28-2.12	0.0001
	Number of organs with metastases (1 vs. >1)	1.14	0.90-1.43	0.2757
	Liver-limited disease (no vs. yes)	0.79	0.62-1.00	0.0486
	Lung-limited disease (no vs. yes)	1.51	0.80-2.84	0.2030
OS2nd	FIRE-3 treatment arm (bevacizumab vs. cetuximab)	0.70	0.55-0.88	0.0024
	ECOG (0 vs. 1 vs. 2)	1.30	1.04-1.62	0.0227
	Age (years)	1.01	0.99-1.02	0.3837
	Sex (male vs. female)	0.93	0.73-1.19	0.5866
	Primary tumor location (left vs. right)	1.55	1.19-2.03	0.0012
	Number of organs with metastases (1 vs. >1)	1.37	1.08-1.74	0.0090
	Liver-limited disease (no vs. yes)	0.72	0.56-0.93	0.0119
	Lung-limited disease (no vs. yes)	0.67	0.38-1.19	0.1736

Cox proportional hazard regression model. CI= confidence interval. All 411 pts included in all factor-analyses, except for “number of organs with metastases”: (2 patients with missing data).

**Table 4:** Multivariate analysis of PFS2nd and OS2nd (backward selection)

Parameter	Factor	Hazard ratio	95% CI	P-value
PFS2nd	FIRE-3 treatment arm (bevacizumab vs. cetuximab)	0.73	0.58-0.91	0.0064
	Primary tumor location (left vs. right)	1.54	1.19-2.00	0.0011
	Liver-limited disease (no vs. yes)	0.78	0.61-0.99	0.0384
OS2nd	FIRE-3 treatment arm (bevacizumab vs. cetuximab)	0.74	0.58-0.94	0.0127
	ECOG (0 vs. 1 vs. 2)	1.30	1.04-1.63	0.0219
	Primary tumor location (left vs. right)	1.45	1.11-1.91	0.0065
	Liver-limited disease (no vs. yes)	0.70	0.54-0.90	0.0055

Cox proportional hazard regression model. Only factors with significant influence on outcome are shown. CI= confidence interval.

In the subset of patients with RAS and BRAF wild-type tumors, similar observations were made. PFS2nd (LPT: 6.1 [95% CI 5.6-6.8] vs. RPT: 5.3 [95% CI 3.3-6.9] months, hazard ratio: 0.72 [95% CI 0.51-1.02], P=0.07) and OS2nd (LPT: 17.3 [95% CI 15.3-20.9] vs. RPT: 12.0 [95% CI 9.9-17.7] months, hazard ratio: 0.58 [95% CI 0.40-0.83], P=0.003) were more favourable in LPT as compared to RPT, respectively. Both in PFS2nd and in OS2nd, this difference based on the initial-cetuximab treated patients in LPTs (hazard ratio in favour of initial cetuximab arm vs. initial bevacizumab arm for PFS2nd: 0.63 [95% CI 0.45-0.89], P=0.007 and for O2nd: 0.58 [95% CI 0.40-0.82], P=0.002). No comparable difference was observed between the initial study arms in RPTs (hazard ratio for PFS2nd: 1.37 [95% CI 0.72-2.58], P=0.33 and for OS2nd: 1.15 [95% CI 0.61-2.17], P= 0.67).

### Interaction of pre-treatment with cetuximab or bevacizumab and primary tumor sidedness in second-line therapy

**Figure 4:**
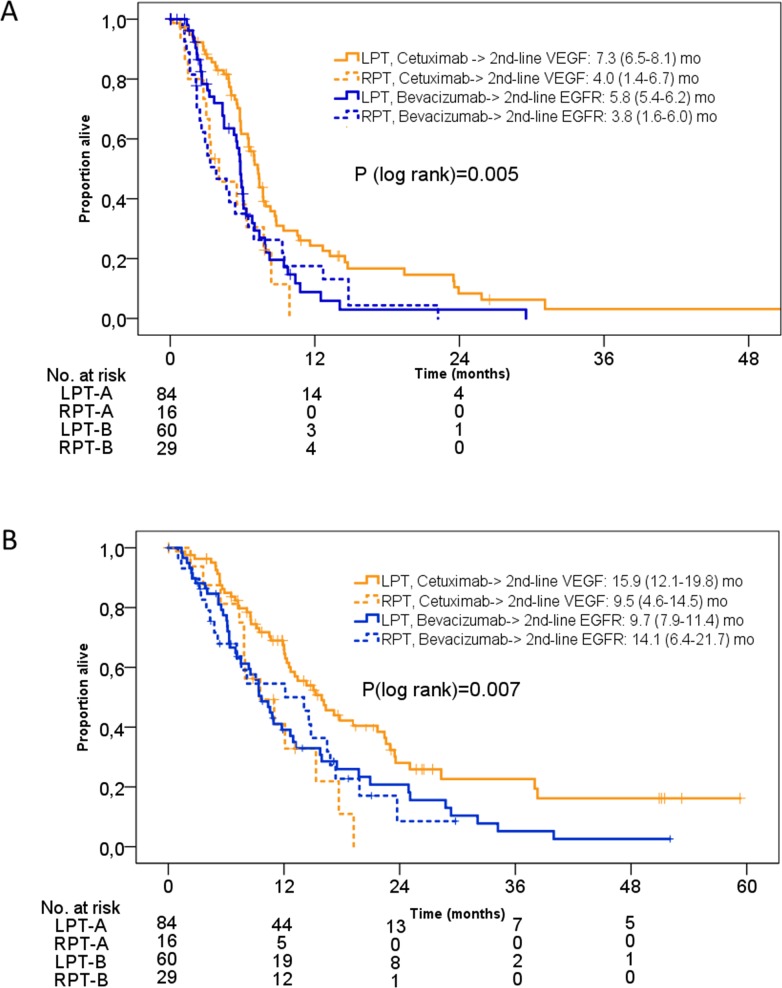
Kaplan-Meier estimates of PFS2nd and OS2nd in the FIRE-3 KRAS exon 2 wild-type population according to antibody-crossover sequences by initial study arm and primary tumor location **(A)** PFS2nd according to tumor location and antibody-sequence; **(B)** OS2nd according to tumor location and antibody-sequence. LPT= left-sided primary tumor, RPT= right-sided primary tumor. A= arm A= initial cetuximab. B= arm B= initial bevacizumab.

We also explored the sequence of cetuximab followed by anti-VEGF antibody versus bevacizumab followed by anti-EGFR antibody with respect to primary tumor location. In patients with left-sided primary tumors cetuximab-followed by VEGF-targeted agents was associated with a more favourable PFS2nd of 7.3 [95% CI 6.5-8.1] months as compared to the reverse sequence (5.8 [95% CI 5.4-6.2] months). This difference was significant: hazard ratio 0.59 [95% CI 0.40-0.88], P= 0.01. In patients with RPT, PFS2nd was 4.0 [95% CI 1.4-6.7] months in patients with initial cetuximab--followed by bevacizumab-based therapy) versus 3.8 [95% CI 1.6-6.0] months in patients receiving the reverse sequence, hazard ratio: 1.04 [95% CI 0.83-1.32], P=0.72. A similar observation was again made for OS2nd. Please see Figure 4 for details.

## DISCUSSION

With primary tumor sidedness attracting increasing attention for decision making in the first-line therapy setting of mCRC, this manuscript aims to explore the role of primary tumor location for treatment efficacy beyond first-line therapy in the context of a randomised first-line trial. The present analysis appears relevant since available data concerning primary tumor sidedness and efficacy of systemic treatment mainly focus on first-line studies [1–5, 13]. Data on pretreated patients are rarely available and may not provide information on the respective sequence [9, 10, 14]. With increasing chances for mCRC patients to experience survival beyond two years, the majority of patients receive at least one further therapy (second-line therapy). Although FIRE-3 was a randomised first-line study, detailed data concerning second-line treatment and its efficacy is available [12]. The continuous observation as used in this manuscript lacks randomisation in second-line therapy, but on the other hand allows observations of second-line treatment effects on the basis of a standardized prior (first-line) therapy.

In FIRE-3, the frequency of second-line therapy was approximately 70%, based on a definition that required the use of an antitumor agent not used in 1st-line. Although the sequence of regimens might have lead to certain imbalances in their composition, the use of available agents appeared balanced between the study arms [15, 16]. If subgroups of treatment arms and primary tumor location are analysed, the highest and lowest rates of second-line therapy were observed both, in the bevacizumab-arm of FIRE-3 with the highest rate (surprisingly) in patients with right-sided mCRC. Interpretation of these data requires caution since multiple other factors may influence these results (secondary resections etc). However, this observation supports the available data on right-sided mCRC treated with bevacizumab-based first-line therapy [1, 4].

Analysis of death-rates according to treatment arm in FIRE-3, primary tumor location and treatment line was performed to understand in which treatment line the death-event was not avoided by treatment. Whereas all combinations of study-arms/primary tumor location were associated with death-rates between 12.8 and 15.8%, patients with RPT in arm A showed a death rate as high as 19% while on first-line therapy. Interestingly, in second-line treatment the highest death rate was observed in patients with RPT after initial bevacizumab-therapy. Of course, in addition to various treatment combinations, multiple co-factors that may not even be known, may bias our observation. Nevertheless, if potential crossover-therapy is taken into account, it occurs that cetuximab-based therapy offers a favourable opening for an algorithm in patiens with LPT, while bevacizumab appears to induce clearly better outcome when applied as initial therapy in patients with RPT (in patients with RAS/BRAF wild-type tumors respectively [17]). The fact, that a considerable number of patients with RPT face death within the early stages of systemic therapy, may stimulate closer follow-up of this high-risk population.

Efficacy of second-line regimens was clearly influenced by primary tumor location and study arm. In general, patients with left-sided mCRC experienced more successful second-line treatment as reflected by PFS2nd and OS2nd. This supports the general prognostic impact of tumor location [1–5, 13, 18]. In FIRE-3, the correlation of second-line treatment efficacy and primary tumor location on might be more direct than in first-line therapy, given that both PFS2nd and OS2nd were affected, which was not the case with first-line PFS [5]. However, this difference appeared primarily to be driven by patients with left-sided tumor from the initial cetuximab-arm. This effect on PFS2nd, also present in OS2nd, might again support the concept that a treatment sequence starting with cetuximab provides a favorable pre-condition for subsequent agents, while there was no clear impact of prior treatment arm in patients with RPT. The smaller sample size in patients with RPT limits firm conclusions from the presently available studies, including this one. Based on the data available from FIRE-3, efficacy of second-line therapy in RPT appeared similar in both initial study-arms with clearly more patients of the initial bevacizumab-arm being exposed to further systemic therapy. A potential explanation of this finding could be that initial cetuximab caused adverse effects in patients with RPT and consecutively prohibited second-line therapy.

To gain a precise insight into distinct sequences of targeted agents, we compared initial cetuximab (arm A) followed by VEGF-targeted therapy with initial bevacizumab (arm B) followed by EGFR-targeted regimens with respect to primary tumor sidedness. Other sequences, including “bevacizumab beyond progression”, were not analysed due to low patient numbers and potential further confounders. Although, this analysis suffers from a limited sample size and the absence of the TML-type strategy (bevacizumab beyond progression) [18], again the greatest benefit was derived from second-line anti-VEGF therapy after cetuximab-based first-line treatment in patients with LPT. This benefit from a defined sequence, as observed in LPT, was not found in RPT. This result is indirectly supported by a large number of second-line trials with clear evidence of benefit from anti-VEGF-based therapy [18–21]. Additionally, there is some increasing evidence that in second-line therapy of mCRC anti-VEGF agents may represent a better choice as compared to EGFR-targeted agents [12, 22, 23].

The main limitation of this manuscript is of course its retrospective nature, the limited sample size in subgroups, and the fact that observation across treatment lines invokes an increasing potential of bias. Although a certain standardisation by first-line therapy might control effects, this analysis does not replace a necessary trial prospectively exploring sequential use of monoclonal antibodies.

In conclusion, our data provide evidence that primary tumor sidedness impacts on outcome of second-line therapy. Besides a purely prognostic information, certain treatment sequences may help to support this effect. Our findings on second-line efficacy and death in several treatment lines support the concept that an optimized treatment algorithm for patients with *RAS* wild-type mCRC and left-sided primary tumors should contain the initial use of an EGFR-targeted antibody, while bevacizumab should preferably be selected as 1^st^-line therapy in mCRC patients with RPT.

## MATERIALS AND METHODS

### Patient population

The second-line population of FIRE-3 was described previously [12]. The collection of data regarding subsequent treatment was predefined in the study protocol. Data were checked for plausibility and monitored by an independent Clinical Research Organisation (CRO). The present manuscript is based on patients with systemic subsequent therapy (i.e. chemotherapy and/or targeted agents) with or without additional modalities (surgery or other interventions). Recruitment of the study lasted until 2012, currently available drugs for the treatment of mCRC, such as regorafenib and TAS-102 were therefore not available for the vast majority of study patients. During the conduct of FIRE-3, fluoropyrimidines, oxaliplatin, irinotecan, cetuximab, and bevacizumab were available to all patients for treatment of refractory mCRC without limitations by sequence of reimbursement regulations. The data cut-off date for this analysis was August 2014.

### Study

FIRE-3 compared FOLFIRI plus cetuximab (arm A) to FOLFIRI plus bevacizumab (arm B) as first-line treatment of mCRC patients with *KRAS* exon 2 wild-type tumors. Consecutively, 414 patients of the total of 592 patients received second-line therapy that appeared to be more effective in patients of the initial cetuximab-arm [12]. Data on second-line efficacy represent retrospective analyses. The responsibilities within the conduct of the trial, as well as the full study population, treatment schedules, Declaration of Helsinki, ethic committee approval and analysis of mutations in *KRAS* exon 2-4 and *NRAS* exon 2-4, *BRAF* as well as evaluation of the second-line population were reported previously [11, 12, 24]. FIRE-3 is registered with ClinicalTrials.gov (NCT00433927).

### Lines of therapy and death in treatment-lines

As described previously [12], second-line therapy was defined as first administration of any anticancer drug that was not included in the first-line regimen. Accordingly, any later-line treatment was defined as use of an anticancer drug that was not part of the prior treatment regimen. Proportion of patient deaths in treatment lines were calculated based on the first-line (study-) population and analysed descriptively. Deaths in treatment lines were evaluated with respect to the last administered treatment-line.

### Primary tumor sidedness

Left-sided primary tumors (LPTs) were defined as those originating from the splenic flexure, descening colon and sigmoid colon, as well as the rectum. Right-sided primary tumors (RPTs) included the coecum, ascending colon, hepatic flexure and colon transversum.

### Progression-free survival (PFS2^nd^), overall survival (OS2^nd^) of second-line therapy

Progression-free survival of second-line therapy was defined as the time from first application (at least one application defined 2^nd^-line therapy) of second-line therapy to disease progression or death from any cause. PFS2^nd^ was evaluated by the local investigator [12]. OS2^nd^ was defined as the time from first application of second-line therapy to death from any cause [12]. In patients without reported progression or death during second-line therapy, respective outcomes were censored to the last reported date of therapy or observation. PFS2^nd^ and OS2^nd^ represent medians.

### Statistical analysis

PFS2^nd^ and OS2^nd^ were analysed by the Kaplan-Meier method and were compared by log-rank tests. Hazard ratios (HRs) were calculated by Cox regression models. Cox proportional hazard regression models were used for multivariate analyses. Differences between groups in dichotomous variables were analysed using two-sided Fisher's exact tests. Two-sided P-values <0.05 were considered significant. All statistical analyses were performed using SAS 9.2 or higher (SAS Institute Inc., Cary, NC, USA) and IBM SPSS Statistics 22 (IBM Corporation, Armonk, NY, USA).
